# Book Review: Current Perspectives on Child Language Acquisition: How Children Use Their Environment to Learn

**DOI:** 10.3389/fpsyg.2021.710903

**Published:** 2021-06-25

**Authors:** Xiaoling Zhang, Xiaoxiang Chen, Fei Chen

**Affiliations:** ^1^School of Foreign Languages, Hunan University, Changsha, China; ^2^Department of Foreign Languages, College of Arts and Sciences, National University of Defense Technology, Changsha, China

**Keywords:** child language acquisition, usage-based, grammatical constructions, levels of variation, language disorders

Caroline F. Rowland, Anna L. Theakston, Ben Ambridge, and Katherine E. Twomey (Amsterdam; Philadelphia, PA: John Benjamins Publishing Company), 2020, 330 pages, ISBN: 9789027207074 (hardback), 9789027261007 (e-book)

For child language development, the nature vs. nurture debate has been ongoing for a long time, but neither side is presumably persuaded by the other side. Nativists assume children are equipped with learning mechanisms for morpho-syntax innately and the lexicon with access to innate linguistic representations (Chomsky, 2011). In contrast, the usage-based theorists claim that language development is the product of language input as well as children's internal learning mechanism which is domain-general (Lieven, 2016). However, as Karmiloff-Smith (1998) mentioned that “all scientists from the staunchest Chomskian nativist to the most domain-general empiricist agree that development involves contributions from both genes and environment.” The main disputes lie in the relative contribution of genes and environment and their interactions. How children interact with the environment in their language development has long fascinated researchers from different language and cultural backgrounds. The book *Current Perspectives on Child Language Acquisition: how children use their environment to learn* is a collection of essays of some researchers who have focused on child-environment interaction, and been influenced by the studies of Elena Lieven (Rowland et al., 2020), to whom this book is dedicated.

The book consists of two parts with a total of 13 essays, 7 in the first part “Levels of Acquisition,” which centers on child-environment interactions across different levels of development. The starting chapter provides readers a review of the early communicative development of infants and some of the theoretical perspectives. Chapter 2 discusses several developmental robotics models of the language acquisition, showing the significance for cross-disciplinary collaboration in future theory development and a rising new outlook of language development in which both non-linguistic and linguistic input triggers language development. Then, the following five chapters (Ch3 to Ch7) elaborate the child-environment interaction in morphosyntactic and semantic acquisition from diverse facets, such as grammatical categorization, transitive-causative overgeneralization errors, construction of form-meaning mappings and complex syntactic structures, and Theory of Mind development, mainly based on the previous research of German and English. Generally speaking, the findings of these research all indicate the significance of language input context.

The second part of the book, “Levels of Variation,” includes 6 essays, which discuss variations across individuals, languages and cultures in language development. Among them, two (Ch8&9) focus on language acquisition of typical developing children. Chapter 8 elaborates a dynamic, interactive explanation of gesture development at prelinguistic stage, in which infants' gestures become social over time via interaction with other more experienced speakers. Chapter 9 illustrates how individual differences provide an essential perspective in the language acquisition from gestures to morphosyntax and proposes three casual factors to explain individual differences. Chapters 10 and 13 introduce a few research into Developmental Language Disorders (DLD) and autism and the implications from usage-based theories of language development. Chapter 11 proposes a maximal diversity method, which samples from structurally diversified languages to avoid sampling bias. Chapter 12 discusses language development in bilingualism by detecting factors that account for children's bilingual experience and its position in the input, the interaction between the processing skills and pragmatic skills, and representations across two languages.

As mentioned in the book's introduction, it's the first attempt to bring some of the new perspectives together in one place. It comprises multiple dimensions of child language development: theories vs. empirical evidence, lexical vs. morphosyntactic development, monolingualism vs. bilingualism, and healthy children vs. children with language disorders and etc. It explores how children make use of collective sources of information from environment, construct linguistic representations at several diverse aspects, and learn how to incorporate these representations to conduct effective communication. These findings have enlightened fresh theoretical perspectives which concentrate more on interpreting learning as a complicated dynamic child-environment interaction.

It would be more comprehensive if the book could include more studies of tone languages. Languages of the world exhibit a natural diversity. It is important to note that the existing theories explaining the child language acquisition are mostly based on findings in children from Romance and Germanic language backgrounds such as English, Spanish, German, French, etc. (Singh and Fu, 2016). Actually, not all theories operate universally regardless of the language background. A natural consequence is that the existing theories explaining child language development may not be generalizable to the tone-language-speaking children.

## Author Contributions

XZ and FC selected the book. XZ drafted the book review. XC and FC provided valuable suggestions and revised it. All authors contributed to the article and approved the submitted version.

## Conflict of Interest

The authors declare that the research was conducted in the absence of any commercial or financial relationships that could be construed as a potential conflict of interest.

## References

[B1] Chomsky N. (2011). Language and other cognitive systems. What is special about language? Lang. Learn. Dev. 7, 263–278. 10.1080/15475441.2011.584041

[B2] Karmiloff-Smith A. (1998). Development itself is the key to understanding developmental disorders. Trends Cogn. Sci. 2, 389–398. 10.1016/S1364-6613(98)01230-3 21227254

[B3] Lieven E. (2016). Usage-based approaches to language development: where do we go from here? Lang. Cogn. 8, 346–368. 10.1017/langcog.2016.16

[B4] Rowland C. F. Theakston A. L. Ambridge B. Twomey K. E. (2020). Current Perspectives on Child Language Acquisition: How Children Use Their Environment to Learn. Amsterdam; Philadelphia, PA: John Benjamins Publishing Company. 10.1075/tilar.27

[B5] Singh L. Fu C. S. (2016). A new view of language development: the acquisition of lexical tone. Child Dev. 87, 834–854. 10.1111/cdev.12512 27007329

